# ^177^Lu-PSMA radioligand therapy of predominant lymph node metastatic prostate cancer

**DOI:** 10.18632/oncotarget.26789

**Published:** 2019-03-29

**Authors:** Finn Edler von Eyben, Aviral Singh, Jingjing Zhang, Karin Nipsch, Danielle Meyrick, Nat Lenzo, Kalevi Kairemo, Timo Joensuu, Irene Virgolini, Cigdem Soydal, Harshad R. Kulkarni, Richard Paul Baum

**Affiliations:** ^1^ Center of Tobacco Control Research, Odense, Denmark; ^2^ Theranostics Center for Molecular Radiotherapy and Molecular Imaging, Zentralklinik Bad Berka, Bad Berka, Germany; ^3^ GenesisCare Oncology, Theranostics, East Freemantle, Australia; ^4^ School of Medicine and Pharmacology, University of Western Australia, Nedlands, Australia; ^5^ Docrates Cancer Center, Helsinki, Finland; ^6^ Department of Nuclear Medicine, University Hospital in Innsbruck, Innsbruck, Austria; ^7^ Department of Nuclear Medicine, University of Ankara, Faculty of Medicine, Universitesi Tip Faultesi Sikkiye, Ankara, Turkey

**Keywords:** metastatic prostate cancer, lutetium prostate specific membrane antigen radiolabeled radionuclide therapy, PSA response, relapse treatment, overall survival

## Abstract

^177^Lu-PSMA radioligand therapy (LuPRLT) is mainly used for patients with metastatic castration-resistant prostate cancer who are resistant to established drugs. This study describes LuPRLT, either LuPSMA I&T or LuPSMA RLT-617, for 45 patients with predominant lymph node metastatic prostate cancer (LNM PC). Thirty-five patients had LNM and ten patients had LNM and one or two bone metastases. Before LuPRLT, the patients had prostate specific antigen (PSA) of median 18 µg/l (interquartile range (IQR): 3.3–39). LuPRLT was given with a cumulative injected ^177^Lu activity of median 14.5 GBq (IQR: 12.2–20.4). Maximum percentage decline of PSA was median 92% (IQR: 70–99). Thirty-five patients with only LNM had a better overall survival (OS) than ten patients with LNM and one or two bone metastases. Thirty-three docetaxel-naïve patients had a longer PSMA PET/CT progression-free survival than twelve patients who were resistant to docetaxel. Twenty-two patients who received LuPRLT with a cumulative injected ^177^Lu activity ≥ 14.8 GBq had a better PSMA PET/CT progression-free survival than 23 patients who received LuPRLT with a lower cumulative injected ^177^Lu activity. Seventeen patients with relapse after LuPRLT who received rechallenge LuPRLT or ActPRLT had a better OS than five patients who received other forms for relapse treatment. LuPRLT gave mild and transitory adverse effects. The findings of the present study suggest that LuPRLT of patients with LNM may be effective and safe. The promising results motivate randomized phase II trials to further quantify the impact of LuPRLT as treatment of patients with LNM.

## INTRODUCTION

There are currently five established life-prolonging treatments of patients with metastatic castration-resistant prostate cancer (mCRPC): abiraterone, enzalutamide, docetaxel, cabazitaxel, and ^223^RaCl_2_ (Xotigo^®^). Since 2013, increasing attention has been given for prostate specific membrane antigen (PSMA) as a tool for diagnosis and treatment. Regarding small molecule PSMA based treatment, the most commonly used Lutetium-177 [177Lu] radioligands are LuPSMA I&T and LuPSMA-617.

Lu PSMA radioligand therapy (LuPRLT, either LuPSMA I&T or LuPSMA-617) has mainly been given to patients with end-stage mCRPC resistant to established drugs [1, 2]. A meta-analysis showed that LuPRLT for patients with end-stage mCRPC gave a better maximum percentile decline of prostate specific antigen (PSA) than third-line treatment with established drugs [3]. Recent studies reported several factors that had a significant impact on the outcome with LuPRLT.

In addition, two studies reported encouraging findings as LuPRLT was given to 20 patients who only had lymph node metastases (LNM) of prostate cancer (PC) [4, 5]. The present study reports a longer follow-up of the 20 patients and expanded the number of patients to a total of 45 patients with predominant LNM with or without one or two bone metastases.

Aims for the present study of LuPRLT were: 1) to report maximum percentile decline of prostate specific antigen (PSA) PSA progression-free survival (PFS), PSMA PET/CT PFS, and overall survival (OS); 2) to report whether LuPRLT for LNM patients who had or did not have one or two bone metastases differed in outcome; 3) to evaluate the impact on outcome of docetaxel-status; 4) to evaluate the impact of cumulative injected ^177^Lu activity and impact of relapse treatment at progression after initial LuPRLT; 5) to report adverse effects; and 6) to evaluate whether Eurasian and Australian centers differed regarding outcomes with LuPRLT.

## RESULTS

### Baseline characteristics and disease history

The enrolled 45 patients started LuPRLT between December 11, 2013, and October 17, 2017. Table 1 shows clinical characteristics of the 45 patients before the start of LuPRLT. Regarding 30 patients evaluable for the diagnostic criteria, 16 patients had the diagnosis of LNM based on histology and 14 patients had the diagnosis of LNM based on PSMA PET/CT findings and the clinical course.

**Table 1A:** Clinical characteristics for LNM patients with or without one or two bone metastase

Timing for characteristics	Clinical characteristics and treatments	All patients (*n =* 45)	Patients with LNM (*n =* 35)	Patients with LNM and one or two bone metastases (*n =* 10)
Findings at diagnosis	Age (years, median, IQR)	61 (57–66)	62 (58–69)	60 (51–64)
	Gleason score 6	1	1	0
	Gleason score 7	17	12	5
	Gleason score 8	11	10	1
	Gleason score 9	7	4	3
	LNM	13	10	3
Initial treatment	RP	29	20	9
	RP with pelvic lymph node dissection	13	9	4
	Radiotherapy	13	13	0
Adjuvant treatment	Radiotherapy	13	13	0
	Adjuvant or neoadjuvant ADT	10	8	2
Number of salvage treatments before LuPRLT (median, range)		2 (1–4)	2 (1–4)	3.5 (3–4)
	Radiotherapy	10	4	6
	Lymph node dissection	3	2	1
	ADT	14	8	6
	Docetaxel	12	7	5
	Abiraterone	10	7	3
	Enzalutamide	3	1	2
Findings at start of LuPRLT	Age (years, median, IQR)	70 (64–75)	72 (65–75)	68 (63–72)
	PSA (µg/l, median, IQR)	18.2 (3.3–39)	18.2 (2.3–39)	19.3 (4.1–49)

Abbreviations: ADT, androgen deprivation therapy; IQR, interquartile range; LNM, lymph node metastases; LuPRLT, ^177^Lu-prostate-specific membrane antigen radioligand therapy; RP, radical prostatectomy.

**Table 1B:** Clinical characteristics for Eurasian and Australian patients

Timing for characteristics	Clinical characteristics and treatments	Eurasian patients (*n =* 30)	Australian patients (*n =* 15)
Findings at diagnosis	Age	61 (56.5–65)	63 (57–74)
	Gleason score 6	1	0
	Gleason score 7	14	3
	Gleason score 8	6	4
	Gleason score 9	3	4
Initial treatment	RP	22	7
	RP and pelvic lymph node dissection	13	0
	Radiotherapy	8	5
Adjuvant treatment	Radiotherapy	4	5
	ADT	10	0
Number of salvage treatments before LuPSMA (median, range)		3 (1–4)	1 (1–2)
	Radiotherapy	10	0
	Lymph node dissection	3	0
	ADT	14	0
	Docetaxel	10	2
	Abiraterone	10	0
	Enzalutamide	3	0
Findings at start of LuPRLT	Age (y, median, IQR)	69 (64–73)	73 (63–77)
	PSA (µg/l median, IQR)	23.5 (4–44)	11 (1.5–41)

Abbreviations as in Table 1A.

Before the start of LuPRLT, PSA recurrence after the initial treatment of PC, radical prostatectomy (RP) or external beam radiotherapy, were treated with androgen deprivation therapy (ADT), abiraterone, enzalutamide, docetaxel, and cabazitaxel. One German patient had been treated with cabazitaxel whereas none of the Australian patients had been given abiraterone. On PSMA PET/CT at the start of LuPRLT, 10 patients had local lesions, 29 had regional LNM, 30 had non-regional LNM, and 10 patients had LNM and one or two bone metastases.

LuPRLT given as LuPSMA-617 to 27 patients and as LuPSMA I&T to 18 patients. LuPRLT was carried out with median 3 cycles (IQR: 2–3 cycles) and a cumulative injected ^177^Lu activity of median 14.5 GBq (IQR: 12.2–20.4). Concomitant with LuPRLT, 12 patients were given ADT and two were given abiraterone.

### Effects of LuPRLT

**Figure 1:**
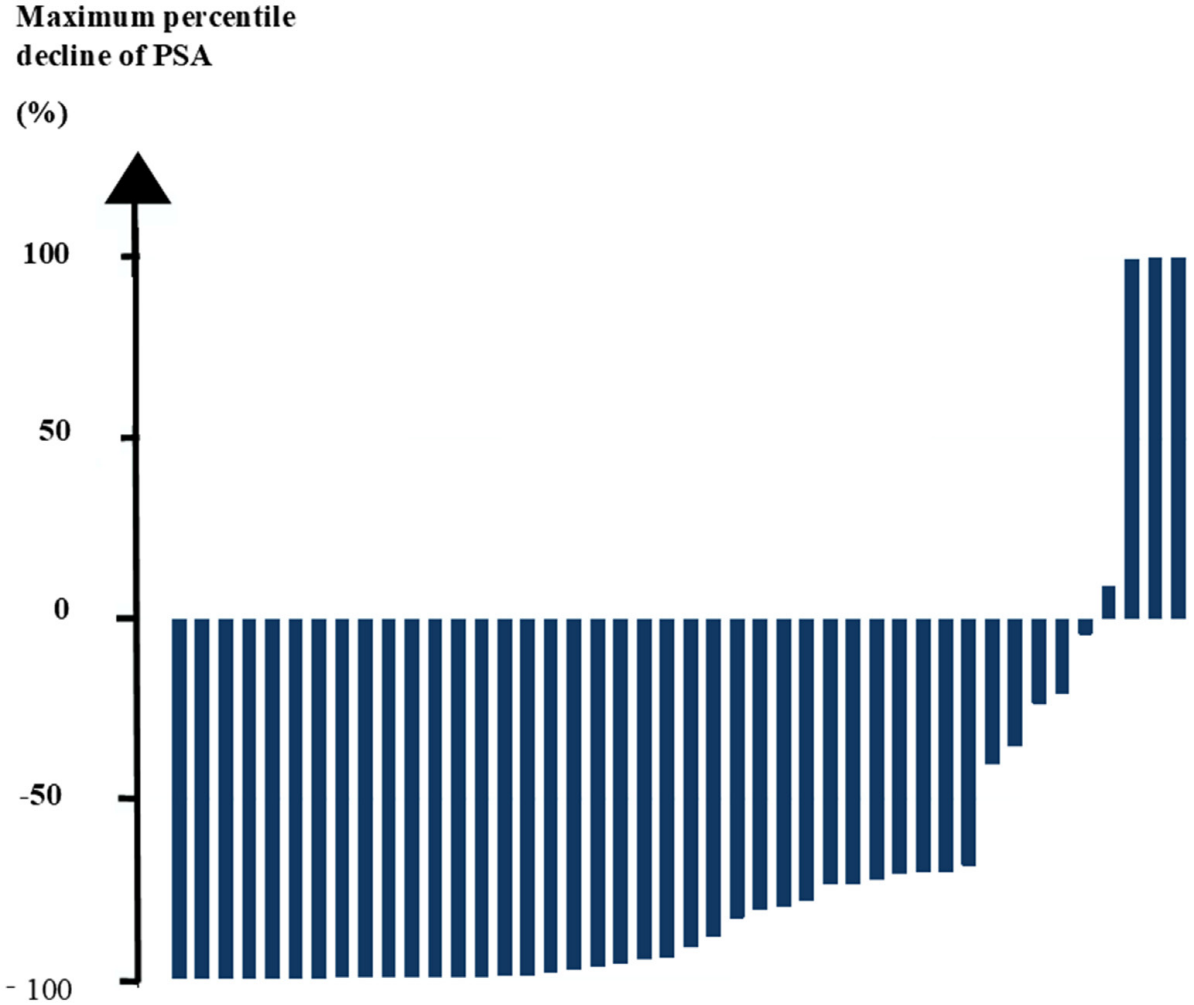
Waterfall plot showing maximum percentile decline of prostate specific antigen (PSA) to ^177^Lu-prostate-specific membrane antigen (LuPSMA) radioligand therapy.

The present study ended follow-up in September/October 2018. Overall, follow-up after LuPRLT was median 26 months [IQR: 18–38]. Three patients (7%) were considered lost at follow-up. Maximum percentile decline of PSA after LuPRLT was median 92% [IQR: 70–99], as shown in Figure 1. Maximum percentile decline of PSA was > 50% for 36 of the 45 patients (80%) and > 90% for 25 patients (56%).

As for the German patients, maximum percentile decline of PSA did not differ significantly between those who had been treated with LuPSMA I&T and those who had been treated with LuPSMA-617 (*p* = 0.84, *t* test).

During follow-up after LuPRLT, 32 patients (71%) had PSA progression. PSA progression-free survival (PFS) was median 16 months. Twenty-five patients developed PSMA PET/CT progression after the baseline series of LuPRLT. At PSMA PET/CT progression, the patients had new sites in only lymph nodes or in both lymph nodes and bones. PSMA PET/CT PFS for Eurasian patients was median 18 months.

**Figure 2:**
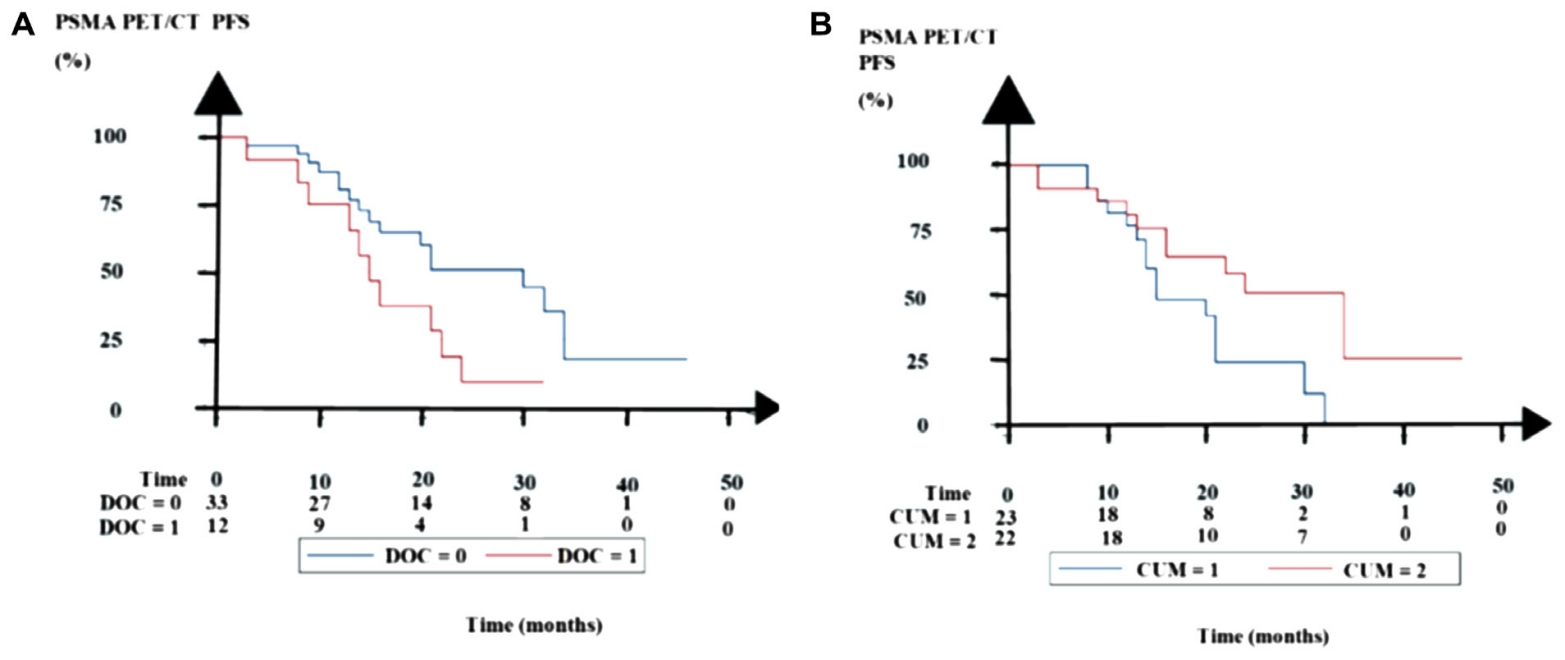
Kaplan–Meier estimates of PSMA PET/CT progression-free survival (PFS) (**A**) Comparison of 33 docetaxel-naïve patient (DOC = 0) and 12 docetaxel-resistant patients (DOC = 1). (**B**) Comparison of 23 patients with median or below median injected cumulative ^177^Lu activity (CUM = 1) with 22 patients with above median cumulative injected ^177^Lu activity (CUM = 2).

Thirty-three docetaxel-naïve patients had a longer PSMA PET/CT PFS than twelve patients resistant to docetaxel (*p* = 0.049, log-rank test, Figure 2A). Twenty-two patients who received LuPRLT with a cumulative injected ^177^Lu activity ≥ 14.8 GBq had a better PSMA PET/CT PFS than 23 patients who received a lower cumulative injected ^177^Lu activity (*p* = 0.03, log-rank test; Figure 2B).

Twenty-two patients with PSMA PET/CT progression after the baseline series of LuPRLT were given salvage treatment. Seventeen relapsing patients were given a rechallenge series of LuPRLT or Actinium based PRLT. Two relapsing patients were given ADT, two enzalutamide, and one chemotherapy. At end of follow-up, two other relapsing patients were followed with active surveillance before they later might be reconsidered for salvage treatment.

**Figure 3:**
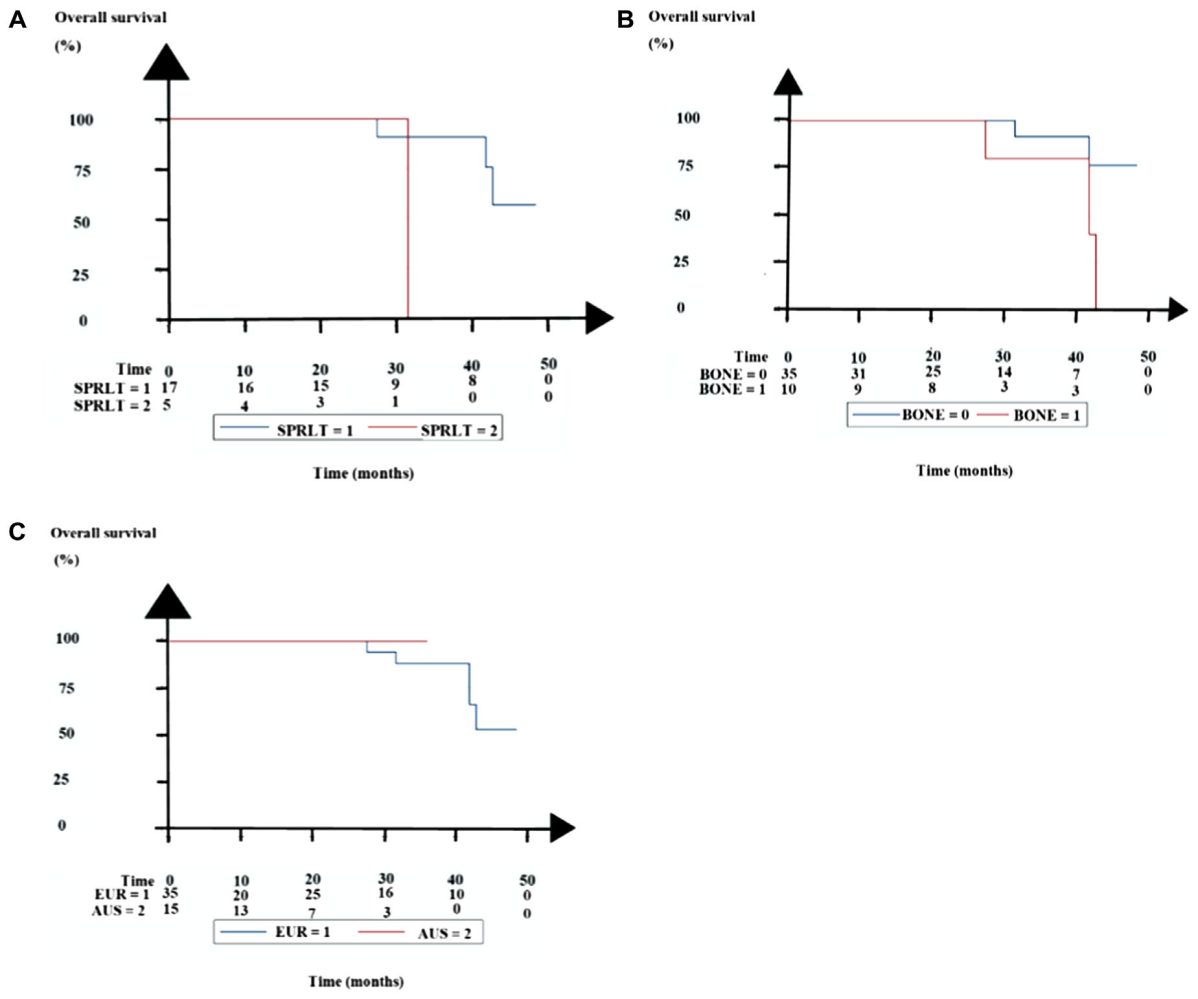
Kaplan–Meier estimates of overall survival (OS) (**A**) Comparison of 17 patients with failure to first series of LuPSMA treatment who were given rechallenge LuPRLT or ActPRLT (SPRLT = 1) and five patients who were given relapse treatment with other drugs than LuPRLT (SPRLT = 2). (**B**) Comparison of 35 patients with only LNM (BONE = 0) and 10 patients with LNM and one or two bone metastases (BONE = 1). (**C**) Comparison of 30 patients treated at four Eurasian centers (EUR = 1) and 15 patients treated at two Australian centers (AUS = 2).

Five patients (11%) died during follow-up. Table 2A and Figure 3 show the overall survival (OS) of the patients. The 17 patients relapsing after baseline LuPRLT who received rechallenge LuPRLT had a better OS than the five patients who received other forms for salvage treatment (*p* = 0.045, log-rank test, Figure 3A).

**Table 2A:** Outcome after ^177^Lu-prostate-specific membrane antigen (LuPSMA) radioligand therapy for patients with LNM with or without one or two bone metastases

Outcomes	Presence of one or two bone metastases		*p*-value
	No (*n =* 35)	Yes (*n =* 10)	
Maximum percentile decline of PSA (%, median IQR)	0.94 (0.74–0.99)	0.54 (–0.09–0.96)	0.016
Median PSA PFS (mo)	17	7	0.21
Median PSMA PET/CT PFS (mo)	27	13	0.35
Median OS (mo)	NR	42	0.046

Abbreviations: IQR, interquartile range; mo, months; NR, not reached; OS, overall survival; PFS, progression-free survival; PSA, prostate specific antigen; PSMA, prostate specific membrane antigen. The *p*-values were based on *t* tests.

### Patients with or without one or two bone metastases

Generally, LNM patients with or without one or two bone metastases had grossly similar clinical characteristics. (Table 1). However, before LuPRLT, the 10 LNM patients with one or two bone metastases had undergone more salvage treatments than the 35 patients with only LNM (*p* = 0.042, *t* test).

Maximum percentile decline of PSA was larger for the 35 patients with only LNM than for the ten patients with LNM and one or two bone metastases (mean 77% vs 19%, *p* = 0.016, *t* test). The 35 patients with only LNM had a good response to LuPRLT, irrespective of the number and regional sites of the LNM. Regarding the 35 patients with only LNM, maximum percentile decline of PSA was > 50% for 31 patients (89%), > 90% for 21 patients (60%) and > 96% for 16 patients (46%).

PSA PFS and PSMA PET/CT PFS did not differ significantly between LNM patients who had or did not have one or two bone metastases. But OS was better for the 35 patients with only LNM than for the ten patients with LNM and one or two bone metastases (*p* = 0.046, log-rank test; Table 2A and Figure 3B).

### Adverse effects

LuPRLT gave mild and transitory adverse effects. None of the 45 patients had severe hematologic or non-hematologic adverse effects. None of the 45 patients were admitted to hospital, stopped LuPRLT, or died due to adverse effects. There were neither reported or observed severe adverse effects. Many Australian patients reported fatigue, and a few Australian patients reported nausea and vomiting.

### Eurasian and Australian patients

Generally, the 30 Eurasian and the 15 Australian patients had grossly similar clinical characteristics and LuPSMA treatment (Table 1B). But the 30 Eurasian patients had undergone more salvage treatments before LuPRLT than the 15 Australian patients (*p* = 0.01, *t* test).

The Eurasian and Australian patients did not differ significantly regarding maximum percentile decline of PSA, PSA PFS, and OS (Table 2B and Figure 3C).

**Table 2B:** Outcome after ^177^Lu-prostate-specific membrane antigen (LuPSMA) radioligand therapy for Eurasian and Australian patients

Outcomes	Eurasian patients	Australian patients	*p*-value
	(*n =* 30)	(*n =* 15)	
Maximum percentile decline of PSA (%, median, IQR)	0.97 (0.70–0.99)	0.88 (0.24–0.96)	0.48
Median PSA PFS (mo)	15	28	0.25
Median PSMA PET/CT PFS (mo)	18	18	0.33
Median OS (mo)	NR	NR	0.63

Abbreviations as in Table 2A.

## DISCUSSION

The present study showed that patients with predominant LNM treated with LuPRLT had a high rate of maximum percentile decline of PSA, high PSA PFS, high PSMA PET/CT PFS, and high OS. Outcomes were better for patients with only LNM than for patients with LNM and one or two bone metastases, for docetaxel-naïve patients than for docetaxel-resistant patients, for patients treated with above median ^177^Lu activity, and for patients given a rechallenge series of LuPRLT at progression after the baseline series of LuPRLT.

These findings supported the promising results indicated in two previous papers [4, 5]. As to a previous case report [4], the present study incorporates an additional 44 patients with predominant LNM. As to a previous report of 19 patients with LNM treated at the Zentralklinik Bad Berka, Germany 2017 [5], the present study reported a longer follow-up to September/October 2018 and added patients treated at an Austrian, a Turkish, and two Australian centers.

All patients in the present study preferred LuPRLT for established drugs and ^223^RaCl_2_. Established drugs for mCRPC had less effect as second-line treatment than as first-line treatment and tended to have more severe adverse effects than LuPRLT. Further, the evidence is weak for supporting third-line treatment with established drugs for patients with mCRPC who are resistant to two established drugs. ^223^RaCl_2_ is not an appropriate treatment for patients with only LNM.

Previously, patients with PSA recurrence after the initial treatment of PC were routinely restaged only with chest roentgenograms and bone scans. Now, restaging with radiolabelled choline PET/CT detects sites of recurrence in lymph nodes for many patients who have PSA recurrence and PSA levels > 2 µg/l. Further restaging PSMA PET/CT detect recurrent sites for more patients than choline PET/CT [6–8]. In addition, progressive use of lymph node dissection in connection with RP may also increase the proportion of patients diagnosed with presence of only LNM.

Patients with regional and non-regional LNM responded to LuPRLT irrespective of the site and number of the LNM. Two previous studies indicated that patients with LNM may have progression of PC only in the lymphatic system [4, 9]. The present study also found that the LNM patients progressed mainly in the lymphatic system. In another study of patients with LNM, circulating tumor cells had a high expression of PSMA [10]. Two systematic reviews showed that PSMA PET/CT had a 75–99% positive predictive value for LNM [11, 12].

The proportion of patients with maximum percentile decline of PSA > 50% was higher for patients with LNM in the present study (31/35 (89%)) than for patients with more advanced mCRPC in other studies from the Zentraklinik Bad Berka, Germany, and the Peter MacCallum Cancer Centre, Melbourne, Australia (46/80 (58%) patients, *p* = 0.001, *χ*^2^ test) [5, 13].

The proportion with maximum percentile decline of PSA > 50% for our patients given LuPRLT for LNM was like that for patients with nonmetastatic castration-resistant PC in another study given first-line abiraterone [14].

PSMA PET/CT was used in the present study to assess objective response to LuPRLT. Studies indicate that PSMA PET/CT may be more sensitive and may detect response to LuPRLT earlier than CT or MRI [2, 5, 15]. In contrast, the Prostate Cancer Clinical Trial Working Group 3 recommended use of conventional imaging modalities to detect PC progression [16]. But restaging PSMA PET/CT may detect progression of PC earlier than conventional imaging. Early detection of relapse may lead to early start of salvage treatments that may benefit the patients.

OS after LuPRLT in the present study was significantly better for the 35 patients with only LNM than for the 10 patients with a combination of LNM and one or two bone metastases. Interestingly, the 10 patient with LNM and one or two bone metastases had a median OS of 41 months. So, the median OS was better than the 13 months (range 8 to 15 months) OS reported in other studies of patients with more advanced mCRPC treated with LuPRLT [13, 17–19].

Taxane chemotherapy-naïve patients with mCRPC had a longer response to LuPRLT than taxane chemotherapy-resistant patients in a previous study [20]. The present study showed the same trend comparing docetaxel-naïve and docetaxel-resistant patients.

Patients who were treated with LuPRLT and received a cumulative injected ^177^Lu activity > 18. 8 GBq had a better survival than patients who received a lower cumulative injected ^177^Lu activity in another previous study [21]. Similarly, the present study found that PSMA PET/CT PFS was better for the patients who had received an above median cumulative injected ^177^Lu activity (≥ 14.8 GBq) than for the patients who had received a lower cumulative injected ^177^Lu activity.

Patients who had PSA recurrence after the baseline LuPRLT and underwent a rechallenge LuPRLT had a better survival than the patients who were treated with other forms for salvage treatments in a third previous study [22]. In the present study, 22 relapsing patients showed the same trend. But only five relapsing patients had been treated with treatments other than LuPRLT.

Hematologic adverse effects in the present study of patients treated with LuPRLT was mild whereas in other studies up to 10% of patients with more advanced multidrug-resistant mCRPC treated with LuPRLT developed severely low hematologic values [22]. The difference might reflect that the patients in the present study had a lower extent of the PC than the patients in the other studies.

Eurasian and Australian centers had similar outcomes regarding maximum percentile decline of PSA, PSA PFS and OS. The similarities in outcomes between the two groups of centers indicate that the findings in the present study appear to have consistency, reproducibility, and internal validity.

The findings of the present study might be better than those reported in other studies of patients with LNM detected with PSMA PET/CT who were treated with salvage lymph node dissection (SLND) [23–26] or salvage external beam radiotherapy (SRT) [27–30]. In those studies, patiens treated with SLND had a median PSA PFS of 4 to 12 months [23, 25, 26]. In our current study, the 35 patients with LNM had a PSA PFS of 16 months despite some of the patients treated with LuPRLT previously having relapsed after SLND and SRT.

An ongoing randomized trial of restaging PSMA PET/CT will recruit 200 patients with PSA recurrence [31]. The trial examines whether PSA PFS will increase if PSMA PET/CT is added to conventional imaging modalities.

Another ongoing randomized trial, Vision (NCT 03511664, www.clinicaltrials.gov), will recruit 700+ patients with end-stage PC. The trial examines whether LuPSMA-617 RLT increases overall survival from 10 months on best supportive care to 13 months on LuPRLT. A third randomized trial, TheraP (ANZUP 1603, NCT03392428), will recruit 200+ patients with abiraterone/enzalutamide- and docetaxel-resistant mCRPC. The trial examines whether LuPSMA-617 RLT gives better outcome than third-line treatment with cabazitaxel.

A fourth small prospective study (NCT03828838) will examined the outcome of LuPRLT for patients with metastatic castration-naïve PC.

The promising results of the present study might be validated in prospective randomized phase II trials. One option for such a prospective randomized phase II trial would be to evaluate the impact of three cycles of LuPRLT before salvage radiotherapy for patients with relapse after an initial RP. Such a prospective randomized phase II trial could be an important supplement to the ongoing trials of LuPRLT that only address patients with more advanced mCRPC.

The present study has strengths. To the best of our knowledge, it is the first study to report OS for patients with LMN treated with LuPRLT. Nearly all available patients in the present study had undergone restaging PSMA PET/CT at the treating centers within the most recent year of follow-up. The present study also had important limitations. It is a retrospective study of a small number of patients followed less than five years.

The findings of the present study suggest that LuPRLT may be effective and safe for patients with LNM. The promising results may motivate randomized phase II trials to further quantify a potential impact of LuPRLT as treatment of patients with LNM.

## MATERIALS AND METHODS

### Setting and study design

Six centers participated in the study. Four centers were Eurasian: i) the Zentralklinik Bad Berka, Badberka, Germany; ii) Docrates Cancer Hospital, Helsinki, Finland; iii) the University Hospital in Innsbruck, Innsbruck, Austria; and iv) the University of Ankara, Ankara, Turkey. Two centers were Australian: v) Hollywood Private Hospital, Nedlands, Western Australia; and vi) Macquarie University Hospital, Macqarie Park, New South Wales, Australia.

The study is a retrospective multicenter single-arm open labelled cohort study.

### Patients

The present study selected patients with PC from prospectively recorded databases of consecutive patients treated with LuPRLT at the six centers. The present study followed conventional eligibility criteria for LuPRLT: informed consent to LuPRLT, age > 18 years, histologically proven PC, hemoglobin > 80 g/l, white blood cell counts > 3 × 10^9^ cells/l, platelet counts > 75 × 10^9^ cells/l, and alanine aminotransferase, aspartate aminotransferase and, with one exception, creatinine < 1.5 × upper limit of normal values [28, 32].

For the present study, additional eligibility criteria were detection of LNM with or without one or two bone metastases, detected with ^68^Ga PSMA PET/CT. The present study included the 20 patients with LNM who had been reported in two previous studies [4, 5], now reported with longer follow-up.

### LuPRLT

Before LuPRLT, all patients were fully informed with respect to the investigational nature of LuPRLT and were made aware of the regulatory status of the investigational drug and of possible and potentially unknown short-term and long-term adverse effects. The patients had been informed that the routine treatment was established life-prolonging drugs. In accordance with the Declaration of Helsinki 1964, all patients had given written informed consent to be treated with LuPRLT before the treatment was instituted.

The Zentralklinik Bad Berka, Germany, treated patients with LuPRLT according to the compassionate clause of the German Medical Product Act. German, Austrian, and Turkish centers adhered to regulations by the German Federal Agency and the Austrian and Turkish Agencies for Radiation protection. The Australian centers adhered to regulations by the Australian Radiation Protection and Nuclear Safety Agency (ARPANSA).

Before each cycle of LuPRLT, the patients underwent a clinical examination. Blood tests included total blood counts, liver and kidney function tests, and PSA measured and reported with 2 decimals. Twenty-seven patients underwent restaging with ^68^Ga-PSMA HBED CC (PSMA) PET/CT and 18 patients were restaged and treated with LuPSMA I&T [33].

The centers first gave LuPRLT as LuPSMA I&T and later as LuPSMA-617. LuPRLT was given as cycles at 8 weeks intervals with an injected ^177^Lu activity of median 6 GBq for each cycle. LuPSMA was injected intravenously over 5–15 minutes. The patients were admitted to hospital for LuPRLT and discharged after 4–6 hours or 2–4 days after the LuPSMA injection, according to local legislation.

For patients responding to LuPRLT, the centers continued to give cycles of LuPRLT until PSMA PET/CT showed no or only small residual lesions. Follow-up after LuPRLT was carried out as follow-up visits at the six centers at 4–12 months intervals. A Turkish patient had a rechallenge series of LuPRLT at another hospital. When follow-up at a Danish university hospital showed PSA recurrence for a Danish patient, he was treated with rechallenge LuPRLT at the Austrian center [3].

### Outcomes

Patients varied regarding PSA response and time to the nadir PSA level after the LuPRLT. The present study calculated the maximum percentile decline of PSA based on the observed nadir PSA. PSA progression was ≥ 25% increase of PSA above the nadir PSA, confirmed with a repeat PSA measurement.

The centers evaluated change in size of lesions according to solid tumor PET response criteria (PERCIST) [29, 33]. In the present study, PSMA PET/CT progression was detection of new or larger metastases on PSMA PET/CT. A lesion was considered to be larger if PSMA PET/CT showed > 25% increase of a diameter of the lesion or > 25% increase of the maximal standardized uptake value (SUV_max_) of the lesion.

PSA PFS was defined as the time from the first day of LuPRLT to PSA progression. OS was defined as the time to end of follow-up or to the death of any cause. The present study considered patients as lost for follow-up if they had not undergone follow-up and restaging at the centers after July 2017.

German and Australian patients reported adverse effects in questionnaires. The treating physicians categorized adverse effects with use of the National Cancer Institute common terminology criteria for adverse effects version 4.0 (CTCAE v 4.0) that considered grade 3 and 4 adverse effects as severe adverse effects.

### Statistical analyses

The statistical analyses evaluated all patients for the observed follow-up. The present study did not substitute missing data. Regarding sample size, the present study selected as many evaluable patients as possible who had been followed at least one year. Regarding comparisons of subgroups of patients, we used *χ*^2^ tests for categorial variables and *t* tests for continuous variables. The present study used Kaplan–Meier estimates and log-rank tests as we estimated survival and compared the survival of subgroups of patients. A *p-*value < 0.05 was considered as statistically significant.

The present study carried out statistical analyses with use of a software program, Stata 14.2 (Stata Corp, College Station, TX, USA).
